# miR-506-3p Relieves Neuropathic Pain following Brachial Plexus Avulsion via Mitigating Microglial Activation through Targeting the CCL2-CCR2 Axis

**DOI:** 10.1159/000528450

**Published:** 2022-12-05

**Authors:** Xing Jin, Wei Zheng, Songyuan Chi, Taihao Cui, Wei He

**Affiliations:** Department of Anaesthesiology, Affiliated Hospital of Beihua University, Jilin, China

**Keywords:** Neuropathic pain, Brachial plexus avulsion, Neuroinflammation, miR-506-3p, CCL2

## Abstract

Neuroinflammation results in neuropathic pain (NP) following brachial plexus avulsion (BPA). This research was designed for investigating the function of miR-506-3p in BPA-induced NP. A total brachial plexus root avulsion model was produced in adult rats as well as IL-1β-treated motoneuron-like NSC-34 cells and the LPS-treated microglia cell line BV2 for in vivo and in vitro experiments, respectively. RT-PCR and Western blot were performed to detect the profiles of miR-506-3p, CCL2 and CCR2, NF-κB, FOXO3a, TNF-α, IL-1β, and IL-6 in cells or the spinal cord close to the tBPI lesion. Neuronal apoptosis was evaluated by immunohistochemistry in vivo. CCK8, TUNEL staining, and the lactic dehydrogenase kit were adopted for the evaluation of neuronal viability or damage in vitro. RNA immunoprecipitation and dual luciferase reporter gene assays analyzed the targeted association between miR-506-3p and CCL2. As shown by the data, miR-506-3p was vigorously less expressed, while CCL2-CCR2, NF-κB TNF-α, IL-1β, and IL-6 were upregulated in the spinal cord with tBPI. Overexpression of miR-506-3p attenuated neuronal apoptosis and microglial inflammation. Mechanistically, CCL2 was a downstream target of miR-506-3p. Upregulating miR-506-3p dampened CCL2-CCR2 and NF-κB activation in the spinal cord and microglia. miR-506-3p had neuroprotective and inflammation-fighting functions in the tBPI rat model via CCL2/CCR2/NF-κB axis.

## Introduction

Brachial plexus avulsion (BPA) is defined as a pre-ganglionic traction injury of the nerve roots from the spinal cord, which involves the severing of axons that form the spinal nerve roots and include the proximal axons of the primary afferent and efferent rootlets [1, 2]. As one of the most serious injuries of the upper extremity, BPA often leads to neuropathic pain (NP), a prevalent and intractable complication [3]. The activation of spinal astrocytes and microglia plays a critical part in the mechanism and treatment of NP [4, 5]. At present, many studies have revealed that suppressing microglial activation elicited by BPA greatly ameliorates NP and neuroinflammation [6, 7]. Therefore, a good understanding of the molecular mechanism of BPA may be important for treating BPA-induced NP.

MicroRNAs (miRNAs), small non-coding RNAs, can repress messenger RNA (mRNA) translation or boost mRNA degradation to modulate gene expression at the posttranscriptional level [8]. Recent studies have verified that miRNAs exhibit aberrant expressions following peripheral nerve injury [9]. For example, the miR-23a level is downregulated in the spinal cord tissues of partial sciatic nerve ligation rats, and miR-23a overexpression hampers CXCR4 to alleviate NP induced by partial sciatic nerve ligation [10]. Subsequent to sciatic nerve chronic constriction injury, miRNA-146a-5p's level is substantially heightened in the L4-L6 DRGs of rats, while miR-146a-5p upregulation can restrain IRAK1 and TRAF6 to mitigate chronic constriction injury-elicited NP [11]. More significantly, in the spinal cords of BPA rats, miR-137-3p expression is downregulated, but miR-137-3p upregulation lowers the level of calpain-2 and the profile of nNOS in spinal motoneurons, hence suppressing neuronal apoptosis induced by BPA [12]. The miR-101-3p level is upregulated in brachial plexus injury rats' spinal cords. JHDM1D-AS1 restrains the profile of miR-101-3p and initiates DUSP1 to hinder neuronal apoptosis and microglial inflammation [13]. miR-506-3p belongs to the miRNA family. It has been extensively investigated in cancer. It is usually used as a cancer-suppressing gene to impede tumor progression [14, 15, 16, 17]. Notwithstanding, the level and role of miR-506-3p under BPA conditions remain unknown.

Monocyte chemoattractant protein-1 (MCP-1) is also named as CCL2 and belongs to the CC family of chemokines. CCR2 is the receptor of CCL2, playing a crucial part in modulating neuroinflammation and NP [18]. Several studies have revealed that in NP incurred by peripheral nerve damage, the profiles of CCL2 and its receptor CCR2 are upregulated in injury models, which is the key to activating spinal microglia [19, 20, 21], whereas CCL2 and CCR2 expression inhibition can attenuate NP induced by nerve damage [21, 22]. Moreover, in the spinal cord tissues of rats suffering from BPA, CCL2 and CCR2 levels increase, while suppressing CCR2 effectively relieves NP elicited by BPA [23]. Given these findings, CCL2 and CCR2 boast significant functions in the context of BPA. On the other side of the fence, classical theories maintain that miRNAs alter the translation efficiency and/or stability of targeted mRNAs to modulate gene expression after transcription [24]. For instance, miR-146a-5p represses TRAF6 and its downstream JNK/CCL2 signal transduction to partly ameliorate SNL-elicited NP [25]. Nonetheless, the relation of miR-506-3p to CCL2 in BPA-induced NP has been less well investigated.

During our research, in vivo and ex vivo assays were made with the use of the adult rat tBPA model, motoneuron-like NSC-34 cells subjected to IL-1β treatment, and BV2 microglia treated with LPS. We discovered that the miR-506-3p level decreased in the ex vivo and in vivo models. miR-506-3p upregulation hampered microglial inflammation and neuronal apoptosis, mitigating NP in BPA rats. Furthermore, miR-506-3p targeted CCL2 and suppressed its profile. As per these outcomes, miR-506-3p might be a target with great potential for treating BPA.

## Materials and Methods

### Cell Culture and Treatment

The Chinese Academy of Sciences (Shanghai, China) was the supplier of NSC-34 neurons and BV2 microglia. A DMEM medium (Thermo Fisher HyClone, UT, USA) supplemented with 10% FBS (Thermo Fisher Scientific, MA, USA) was used to cultivate the cells in an incubator with 5% CO_2_ at 37°C. The solution was changed once every 2 days. The cells were passed once every 5 days. More tests were taken until the cells covered about 90% of the bottle bottom. NSC-34 and BV2 cells, inoculated into 6-well plates (4.0 × 10^5^ cells/mL), were incubated with 100 μg/L IL-1β and 100 ng/mL LPS, respectively, for 24 h. Twenty-four hours later, we examined the cells' viability and apoptosis.

### Cell Transfection

NSC-34 and BV2 cells were seeded onto 6-well plates, and each well contained 4 × 10^5^ cells. 24–48 h later, we transfected the control vector (Vector), CCL2 overexpression plasmids (CCL2), miR-506-3p mimics, or miR-NC (GenePharma, Shanghai, China) into NSC-34 and BV2 cells as per the instructions of Lipofectamine 2000 transfection reagent. Following 48-h incubation, the cells were harvested, and quantitative reverse transcription-polymerase chain reaction (qRT-PCR) was performed to check RNA expression.

### Lactic Dehydrogenase Examination

In keeping with the supplier's recommendations, we employed the lactic dehydrogenase (LDH) cytotoxicity detection kit (Nanjing Jiancheng Institute of Biological Engineering, Nanjing, China) to assess LDH release in the injured neurons for cytotoxicity evaluation. The SpectraMAX340 reader (Molecular Devices, Sunnyvale, CA, USA) measured the value of absorbance (490 nm).

### CCK8 Assay

NSC-34 cells stably transfected were inoculated onto 96-well plates (density: 1 × 10^3^ cells/well) for 24-h incubation. In accordance with the supplier's instructions, each well was given 10 μL CCK8 (Dojindo Molecular Technologies, Kumamoto, Japan). The cells were kept in an incubator for 1 h (37°C). Then, a spectrophotometer (Bio-Rad, CA, USA) immediately confirmed the value of OD450.

### TUNEL Staining

The TUNEL apoptosis detection kit (Keygen BioTECH, Nanjing, China) measured NSC-34 cell apoptosis. An immunostaining fixative immobilized the cells for 30–60 min. PBS flushed them once. Next, the cells were subjected to 2-min incubation with an immunostaining detergent in an ice bath. The solution for TUNEL detection (50 μL) was added, followed by 60 min of incubation in a dark environment at 37°C. PBS rinsed the cells 3 times. An anti-fluorescence quenching sealing liquid was exploited for sealing. A fluorescence microscope was utilized for observation. The excitation light: 450∼500 nm; the emission light: 515∼565 nm (green fluorescence). We randomly selected five fields from each sample. The formula for calculating the rate of apoptosis is as follows: apoptosis rate = apoptotic cells/total cells × 100%.

### Real-Time Quantitative Reverse Transcription PCR

As per the manufacturer's protocol, the total RNA was extracted from tissues or cells employing TRIzol reagent (Invitrogen, Waltham, MA, USA). NanoDrop spectrophotometer determined the concentration and purity of the RNA. As suggested by the manufacturer, we utilized the PrimeScript-RT Kit (Madison, WI, USA) for the synthesis of complementary DNA from 1 μg total RNA. Next, the SYBR®Premix-Ex-Taq^TM^ (Takara, TX, USA) and ABI7300 systems were introduced for qRT-PCR. The PCR system had a total volume of 30 μL. Each sample encompassed 300 ng complementary DNA. We implemented the amplification procedure as follows: 10 min of pre-denaturation at 95°C, followed by 45 cycles, to wit 10 s at 95°C, 30 s at 60°C, and 20 s at 85°C. Next, the fluorescence statistics were converted into relative quantification. GAPDH was taken as the internal parameter of CCL2 and CCR2, TNF-α, IL-1β, and IL-6, whereas U6 was adopted as that of miR-506-3p. All qRT-PCR reactions were duplicated three times. Table 1 details the sequences of the primers we adopted.

### Western Blot

After the cells and tissues were treated, the culture medium was removed. The protein lysis buffer (Roche) separated the total protein. Then, 50 g total protein went through 2 h of electrophoresis (100 V) on 12% polyacrylamide gel. The protein was transferred to polyvinylidene fluoride membranes (Millipore, Bedford, MA, USA). Moreover, 5% skimmed milk powder sealed the membranes for 1 h (RT). TBST flushed them 3 times (10 min each). The primary antibodies we employed for overnight incubation at 4°C encompassed anti-CCL2 (Abcam, ab214819, 1:1,000, MA, USA), anti-CCR2 (Abcam, ab273050, 1:1,000), anti-NF-κB (Abcam, ab32536, 1:1,000), anti-pNF-κB (Abcam, ab76302, 1:1,000), anti-FOXO3a (Abcam, ab109629, 1:1,000), anti-FOXO3a (Abcam, ab154786, 1:1,000), anti-pFOXO3a (Abcam, ab154786, 1:1,000), anti-Bad (Abcam, ab32445, 1:1,000), anti-Bax (Abcam, ab32503, 1:1,000), anti-cleaved caspase-3 (Abcam, ab13585, 1:1,000), anti-iNOS (Abcam, ab178945, 1:1,000), anti-COX2 (Abcam, ab179800, 1:1,000), and anti-GAPDH (Abcam, ab9485, 1:1,000). Subsequent to a wash in TBST, the anti-rabbit secondary antibody labeled by horseradish peroxidase (concentration: 1:3,000) was given for 1-h incubation at RT. TBST rinsed the membranes 3 times (10 min each). In the end, color and image development was done with the use of the reagent of Western blot (Invitrogen). Image J analyzed each protein's gray value.

### ELISA

The ELISA Ready-Set-Go kit (eBioscience, San Diego, USA) gauged the profiles of interleukin-1β (IL-1β), interleukin-6 (IL-6), and tumor necrosis factor-α (TNF-α) in BV2 cells and the spinal cord tissues of BPA rats in conformity with the supplier's instructions. Each experiment was done in triplicate and duplicated twice to evaluate the consistency of outcomes.

### Dual Luciferase Assay

Both luciferase reporter vectors (CCL2-WT, CCL2-mut) were provided by Promega (Promega, Madison, WI, USA). NSC-34 and BV2 cells (4.5 × 10^4^) were inoculated into 48-well plates and cultivated to 70% confluence. Next, the cells were transfected together with CCL2-WT or CCL2-mut and miR-506-3p mimics or the negative control with the use of liposome 2000. Subsequent to 48-h transfection, we examined the luciferase activity in line with the recommendations of the manufacturer. These tests were implemented in triplicate and repeated 3 times.

### RNA Immunoprecipitation Assay

The binding relation of miR-506-3p to CCL2 was substantiated through RNA immunoprecipitation (RIP) analysis, coupled with the Magna RIP Kit (Millipore). Put simply, NSC-34 and BV2 cells were lysed. Agarose beads (Bio-Rad, Hercules, CA, USA), pre-coated with Argonaute-2 antibody (anti-Ago2), were given for incubating the cell lysates. The antibody of immunoglobulin G (anti-IgG) was employed as the control of our research. Later, the RNA samples combined with the beads were separated and purified. qRT-PCR was carried out to check the abundance of miR-506-3p and CCL2 in immunoprecipitation compounds.

### Animals

Forty Sprague-Dawley rats (male or female, 7–8 weeks of age, 230–280 g in weight) were ordered from the Animal Experiment Center of Jilin University. The animals were raised under a 12-h light/dark cycle (20 ± 2°C), allowed to access food and water at any time. This research had received the imprimatur from the Ethics Committee of the Affiliated Hospital of Beihua University. The experiments on animals were implemented in keeping with the guidelines for the management and use of local experimental animals as well as the *Guidelines for the Care and Use of Laboratory Animals* (published by the National Institutes of Health).

### Animal Surgery

The tBPA was performed as suggested by previous studies [26, 27, 28]. In brief, we first confirmed the right brachial plexus (BP), separated cervical nerves (C5, C6, C7, C8, and T1), and pulled out the C5-T1 nerve roots using microscopic pliers close to the root of the near end. The hemostatic forceps, connected with a weight (1.5 kg), were used for pulling the distal parts of the nerves to produce a sudden and powerful force. We excised the extracted anterior and posterior roots and the dorsal root ganglion with a view to guarding against reinnervation. A microscope was adopted to check whether the operation was successful. In the sham operation group, the C8-T1 nerve roots were exposed without damaging the nerves. Ultimately, the incisions were closed layer by layer with 3/0 silk suture, and the local, intramuscular injection of penicillin (5 × 10^4^ U/0.2 mL) into the wounds was conducted for 3 days running (once a day) to prevent infections. Within 15 min after the animals were injured, a 30-gauge needle was utilized to inject miR-506-3p agomir (0.5 nmol; GE Dharmacon, Lafayette, CO, USA) or the negative control agomir (equal concentration; GE Dharmacon) into the left ventricle of the rats at the speed of 0.5 mL/min, in accordance with the method of Henry et al. [29].

### Von Frey Filament Test and Hargreaves Test

In keeping with the approach of Ling et al. [30], von Frey (vF) filaments (Stoelting, Wood Dale, IL, USA) were utilized to evaluate the mechanical threshold of the right anterior paws of all rats on day 1, 4, 7, 14, 21, and 28 following surgery. The animals were placed in a box on the elevated metal mesh floor. A baseline test was conducted. We let the animals adapt to the environment for 30 min prior to examination. A series of vF filaments with logarithmic increasing stiffness (0.02–2.56 g, Stoelting) were taken to stimulate the plantar surface of the right posterior paw. The stimulation lasted for 4 s, with an interval of 30 s. The up-and-down method of Dixon was harnessed to verify the 50% claw threshold. As for the Hargreaves test, the animals were put in a plastic box on a glass plate. The plantar surface was exposed to radiation beams via the transparent glass. The baseline delay was adjusted to 10–14 s, with a maximum of 20 s regarded as the cut-off time to guard against the underlying damage. All experiments were done by people who did not know the detailed treatment of the rats.

### Terzis Grooming Test (TGT)

As per the method of Inciong et al. [31], we performed the TGT on the rats before surgery and on day 1, 4, 7, 14, 21, and 28 after surgery. A syringe was adopted to transfuse 3 mL of water into the nasal cavity of the rats. Then, we observed the impact of clearing water drops in the rats' nasal cavities. The levels range from 0 to 5: 0 refers to no reaction; 1 refers to rats that can bend their elbows but cannot reach their noses; 2 refers to rats that can bend their elbows and touch their noses; 3 refers to rats that can bend their elbows below their eyes; 4 refers to rats that can bend their elbows to the eyes; 5 refers to rats that can bend their elbows near or behind their ears.

### Immunohistochemistry

During the 4 weeks after the BPA model was produced, 5 rats were selected at random from each group and sacrificed under anesthesia through chloral hydrate. The spinal cord tissues surrounding brachial plexus injuries were kept in the paraformaldehyde solution (100 g/L) and fixed for 24 h (RT). The coronal slices (around 3∼4 μm) of the spinal cord tissues were kept for 4–6 h in an oven. PBS flushed the sections 3 times (2 min each). Later, rabbit anti-GFAP (Abcam, ab7260, 1:1,000), anti-IBA1 (Abcam, ab178846, 1:2,000), and anti-cleaved caspase-3 (Thermo Fisher, PA5-114687, 1:200) were added for overnight incubation at 4°C. The following day, the goat anti-rabbit secondary antibody labeled by biotin was taken to incubate the slices for 20 min at RT. DAB was harnessed for color development. Subsequent to sealing, an optical microscope was utilized for observation. The criteria for the outcomes of immunohistochemistry are as follows: at the magnification of ×400 and with five fields chosen at random from each section, the outcomes were regarded as positive if the nuclei were stained pale brown or both the nuclei and cytoplasm were stained pale brown. The scoring of the staining outcomes of positive cells (a): 0 point for <5%, 1 point for 5%∼25%, 2 points for 26%∼50%, and 3 points for >50%. The positive intensity (b): 0 for no color or faint yellow consistent with the background, 1 point for light yellowish brown, 2 points for pale brown, and 3 points for brown. The scoring was done as per the integral of the two indicators (integral = a × b): 2∼3 points for weakly positive (+), 4∼6 for positive (++), and 7∼9 for strongly positive (+++). We worked out and contrasted the mean values of positive and strongly positive cells in each group.

### Statistical Analysis

SPSS17.0 (SPSS Inc., Chicago, IL, USA), a software for statistical analysis, analyzed our data. The diagrams were drawn through GraphPad Prism 8.3 (San Diego, CA, USA). The measurement statistics were exhibited as mean ± standard deviation (X±SD). One-way ANOVA and *t*test contrasted two or more groups. If the *p* value was below 0.05, statistical significance was confirmed.

## Results

### miR-506-3p Expression Was Downregulated in NSC-34 Cells Treated with IL-1β and Microglia Treated with LPS

**Fig. 1:**
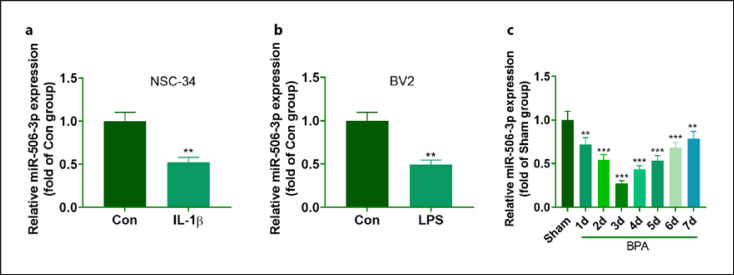
miR-506-3p profile was lowered in IL-1β-treated NSC-34 cells and LPS-treated microglia. The miR-506-3p level in IL-1β-elicited NSC-34 cells (**a**) and LPS-treated microglia (**b**) determined through qRT-PCR. The data were exhibited as mean ± SD (*n* = 3). **p < 0.01 (vs. Con). **c** miR-506-3p expression in the spinal cord tissues of SD rats at different time points from day 1 to day 7 subsequent to BPA surgery examined via qRT-PCR. The data were represented as mean ± SD (*n* = 3). **p < 0.01,***p < 0.001 (vs. Sham).

For exploring the role of miR-506-3p in BPA, we carried out qRT-PCR to confirm the profile of miR-506-3p in NSC-34 cells treated with IL-1β (100 μg/L) and microglia treated with LPS (100 ng/mL). Consequently, the miR-506-3p level decreased in the treated cells (vis-à-vis Con) (Fig. 1a, b). Next, qRT-PCR checked miR-506-3p profile in the spinal cord tissues of the rats at different time points from day 1 to day 7 following surgery. By contrast to the Sham group, the profile of miR-506-3p declined in the spinal cord tissues of the rats with BPA, and its expression hit the lowest on day 3 after injury and gradually increased after day 3, but its level remained lower than that in the Sham group (Fig. 1c). These phenomena unraveled that miR-506-3p might play a significant part in BPA pathogenesis.

### miR-506-3p Suppressed Inflammation in Microglia Induced by LPS

**Fig. 2:**
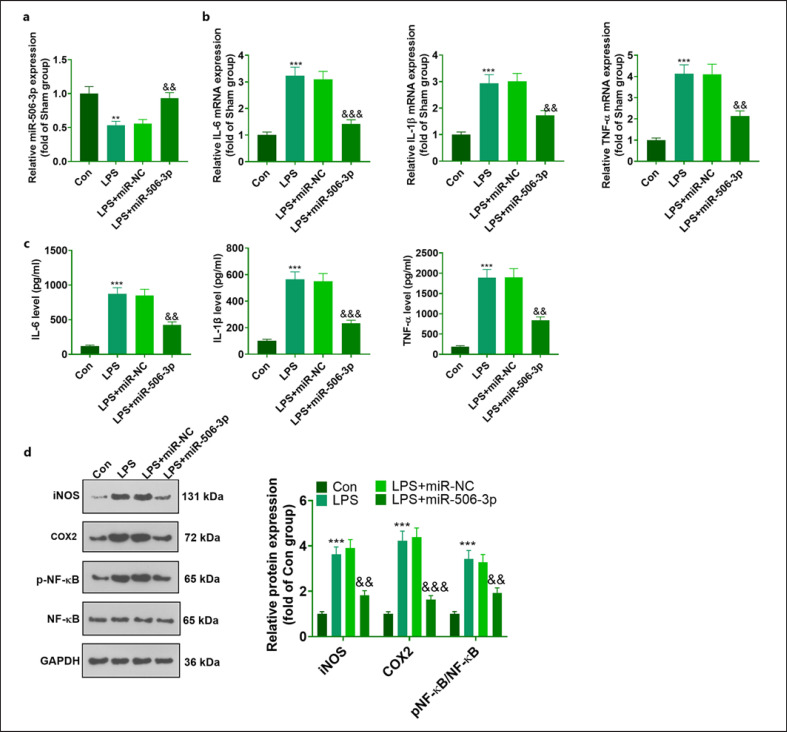
miR-506-3p hampered inflammation in microglia induced by LPS. miR-NC and miR-506-3p mimics were harnessed to transfect LPS-elicited BV2 microglia for 24 h. **a** The miR-506-3p level in BV2 checked by qRT-PCR. TNF-α, IL-1β, and IL-6 levels in the cells confirmed through qRT-PCR (**b**) and ELISA (**c**). **d** iNOS, COX2, and pNF-κB profiles in the cells checked by Western blot. The data were exhibited as mean ± SD (*n* = 3). **p < 0.01,***p < 0.001 (vs. Con). ^&&^*p <* 0.01, ^&&&^*p <* 0.001 (vs. LPS + miR-NC).

To investigate the influence of miR-506-3p on microglia, we transfected miR-NC and miR-506-3p mimics into LPS-elicited BV2 cells. Given the outcomes of qRT-PCR, miR-506 transfection augmented the miR-506-3p level in BV2 microglia induced by LPS, which demonstrated the success of the transient transfection (Fig. 2a). Moreover, qRT-PCR and ELISA suggested that as compared with the Con group, LPS heightened the profiles of inflammatory cytokines (TNF-α, IL-1β, and IL-6) in BV2 microglia. After miR-506-3p mimics were transfected, there was a substantial reduction in TNF-α, IL-1β, and IL-6 levels in BV2 microglia (Fig. 2b, c). The experiment of Western blot reflected that LPS augmented iNOS and COX2 levels and elicited the phosphorylation of NF-κB in BV2 (vs. Con). Subsequent to miR-506-3p mimics transfection, iNOS and COX2 profiles were vigorously lowered, whereas the phosphorylation level of NF-κB was considerably attenuated in BV2 (Fig. 2d).

### miR-506-3p Promoted the Survival of IL-1β-Elicited NSC-34 Neurons

**Fig. 3:**
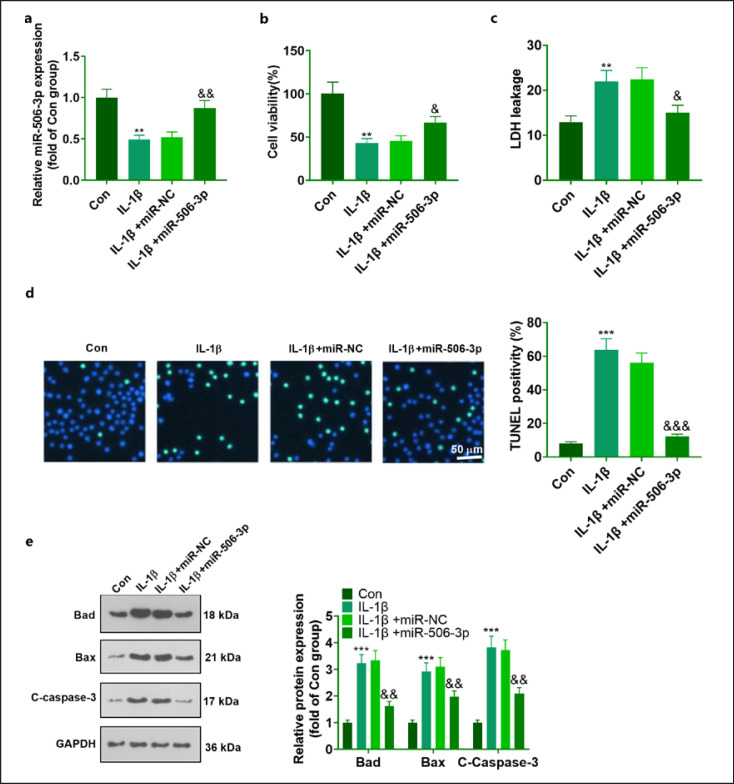
miR-506-3p promoted the survival of motoneurons elicited by IL-1β. With NSC-34 cells as the model, IL-1β was adopted to elicit neuronal damage for simulating an ex vivo model of BPA. NSC-34 cells induced by IL-1β were transfected with the use of miR-NC and miR-506-3p mimics for a culture of 24 h. **a** miR-506-3p profile in NSC-34 determined via qRT-PCR. **b** Cell viability monitored via CCK8. **c** Cell viability evaluated through LDH release. **d** Positive TUNEL cells in the cells counted by TUNEL staining. **e** The profiles of apoptotic proteins verified via Western blot. The statistics were exhibited as mean ± SD (*n* = 3). **p < 0.01, ***p < 0.001 (vs. Con). ^&^*p <*0.05, ^&&^*p <*0.01 (vs. IL-1β + miR-NC).

NSC-34 cells elicited by IL-1β were transfected together with miR-NC and miR-506-3p mimics for a culture of 24 h. The assay of qRT-PCR displayed that transfecting miR-506-3p mimics caused an increase in the miR-506-3p level in the cells elicited by IL-1β, which substantiated the success of the transient transfection (against IL-1β + miR-NC) (Fig. 3a). Moreover, CCK8, LDH, and TUNEL staining suggested that by contrast to the Con group, IL-1β dampened proliferation, bolstered the release level of LDH and the number of positive TUNEL cells in the neurons. By contrast, miR-506-3p overexpression expanded cell proliferation (Fig. 3b) and suppressed LDH release and apoptosis in the cells (Fig. 3c, d). Given the findings of Western blot, it was unveiled that IL-1β elevated the levels of Bax, Bad, and C-caspase-3 (proteins that boost apoptosis) in the neurons. Nevertheless, miR-506-3p upregulation repressed the increase in apoptotic proteins in IL-1β-elicited neurons (Fig. 3e).

### CCL2 Was a Direct Target Gene of miR-506-3p

**Fig. 4:**
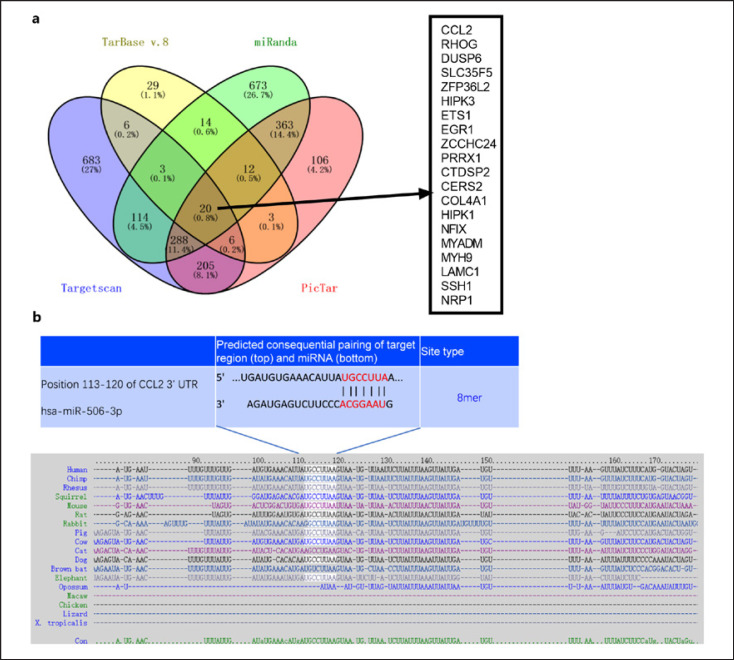
CCL2 was directly targeted by miR-506-3p. **a** The targets of miR-506-3p predicted via Targetscan, Tarbasev8, PicTar, and miRanda. The sharing targets in the four databases analyzed via Venny's diagram (https://bioinfogp.cnb.csic.es/tools/venny/index.html). **b** The binding sites between CCL2 mRNA and miR-506-3p in different species.

**Fig. 5:**
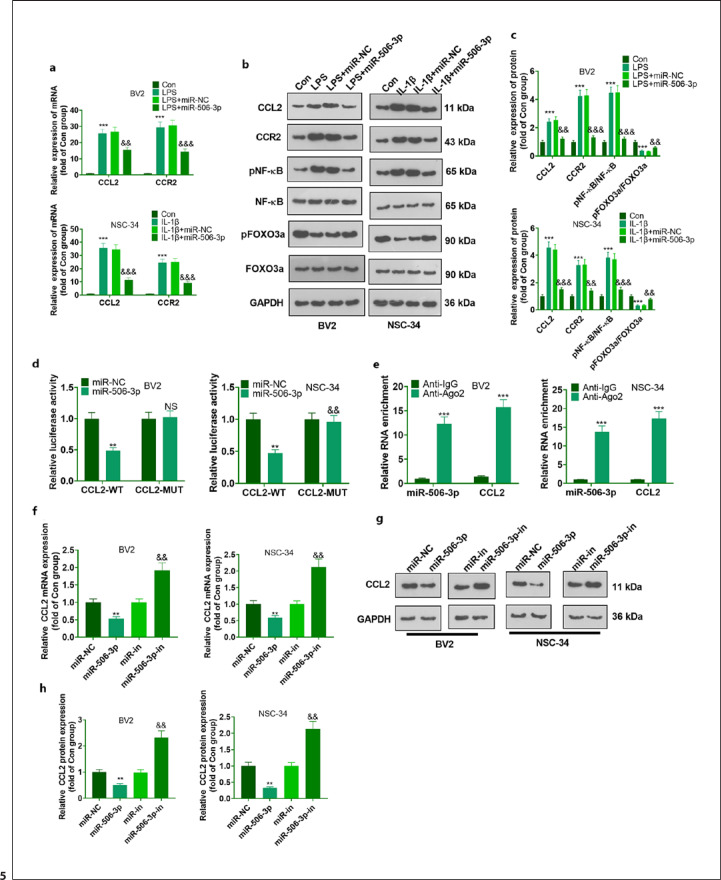
CCL2 was directly targeted by miR-506-3p. CCL2 and CCR2, NF-κB, and FOXO3a levels in LPS-elicited BV2 microglia and IL-1β-elicited NSC-34 neurons determined via qRT-PCR (**a**) and Western blot (**b, c**). **p < 0.01,***p < 0.001 (vs. Con). ^&^*p <* 0.05, ^&&^*p <* 0.01 (vs. LPS + miR-NC and IL-1β + miR-NC). Whether miR-506-3p directly combined with CCL2 confirmed by dual luciferase assay (**d**) and RIP (**e**). **p < 0.01, NSP *>*0.05 (vs. miR-NC); ***p < 0.01 (vs. anti-IgG). **f**−**h** The construction of the cell lines of miR-506-3p overexpression and low expression. The relative profile of CCL2 gauged by qRT-PCR (**f**) and Western blot (**g**−**h**). **p < 0.01 (vs. miR-NC), ^&&^*p <* 0.01 (vs. miR-in). The data were represented as mean ± SD (*n* = 3).

The downstream target of miR-506-3p was predicted using four online websites, including Targetscan (https://www.targetscan.org/vert_72/), Tarbasev8 (https://dianalab.e-ce.uth.gr/html/diana/web/index.php?r=tarbasev8), PicTar: (http://www.pictar.org/), and miRanda (http://www.microrna.org/microrna/home.do). Twenty genes were identified as sharing targets in the four databases using Venny's diagram (https://bioinfogp.cnb.csic.es/tools/venny/index.html) (Fig. 4a). CCL2 is one of the targets among the 20 genes, and the binding sites between CCL2 mRNA with miR-506-3p were shown (Fig. 4b). qRT-PCR and Western blot confirmed that the profiles of CCL2 and CCR2 were heightened in LPS-induced BV2 cells and IL-1β-elicited NSC-34 cells (in contrast with the Con group), whereas miR-506-3p upregulation inhibited their levels (Fig. 5a-c). To verify whether miR-506-3p directly targeted the 3′-UTR of CCL2, dual luciferase reporter gene assay was done. miR-506-3p mimics vigorously attenuated the luciferase activity of CCL2-WT but barely influenced that of CCL2-MUT in BV2 and NSC-34 cells (Fig. 5d). Furthermore, in the immunoprecipitation compounds pulled down by Ago2 antibody, the levels of CCL2 and miR-506-3p were very high, suggesting that CCL2 could combine with miR-506-3p in BV2 and NSC-34 cells (Fig. 4e). Moreover, we produced the miR-506-3p overexpression and low expression cell lines in BV2 and NSC-34 cells. qRT-PCR and Western blot data transpired that the relative profile of CCL2 declined significantly in the miR-506-3p mimic cell line but greatly increased in the miR-506-3p inhibitor cell line (Fig. 5f-h). These phenomena corroborated that miR-506-3p targeted CCL2, and they were negatively correlated.

### miR-506-3p Attenuated LPS-Induced Microglial Inflammation and IL-1β-Elicited Neuronal Apoptosis via the CCL2-CCR2 Axis

**Fig. 6:**
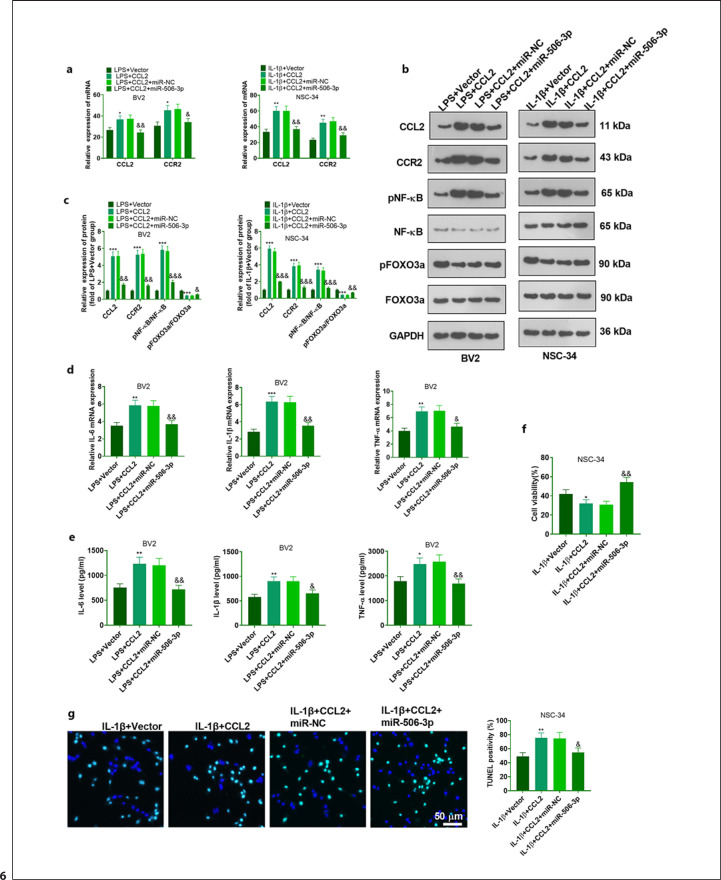
miR-506-3p attenuated LPS-elicited microglial inflammation and IL-1β-elicited neuronal apoptosis via the CCL2-CCR2 axis. LPS-induced BV2 cells and IL-1β-elicited NSC-34 cells were transfected with Vector, CCL2, CCL2 + miR-NC, and CCL2 + miR-506-3p for 24 h of culture. CCL2, CCR2, NF-κB, and FOXO3a profiles in BV2 and NSC-34 analyzed via qRT-PCR (**a**) and Western blot (**b, c**). The levels of inflammatory cytokines in BV2 microglia checked by qRT-PCR (**d**) and ELISA (**e**). **f** NSC-34 cell viability tracked through CCK8. **g** The number of positive TUNEL cells in NSC-34 neurons checked by TUNEL staining for the assessment of apoptosis. The data were exhibited as mean ± SD (*n* = 3). *p < 0.05,**p < 0.01,***p < 0.001 (vs. LPS + Vector; vs. IL-1β + Vector). ^&^*p <* 0.05, ^&&^*p <* 0.01, ^&&&^*p <* 0.001 (vs. LPS + CCL2 + miR-NC; vs. IL-1β + CCL2 + miR-NC).

The above findings revealed that miR-506-3p targeted CCL2. Nevertheless, the functions of miR-506-3p and CCL2 in the context of BPA remained obscure. Therefore, BV2 and NSC-34 were transfected together with Vector, CCL2, CCL2 + miR-NC, as well as CCL2 + miR-506-3p. qRT-PCR and Western blot assays reflected that transfecting CCL2 overexpression plasmid dramatically heightened CCL2 and CCR2 expressions, strengthened NF-κB activation, and repressed FOXO3a activation in LPS-elicited BV2 cells and IL-1β-elicited NSC-34 cells. miR-506-3p upregulation lessened the upregulation of the above mRNA and protein caused by CCL2 overexpression (Fig. 6a-c). qRT-PCR and ELISA assessed the profiles of inflammatory cytokines in BV2 microglia elicited with LPS. The outcomes indicated that in contrast with the LPS + Vector group, CCL2 upregulation remarkably heightened the profiles of inflammatory factors in the LPS-induced cells. Overexpressing miR-506-3p attenuated the profiles of inflammatory factors (Fig. 6d, e). Next, CCK8 and TUNEL staining monitored neuronal viability and apoptosis. By contrast to the IL-1β + Vector group, CCL2 lessened NSC-34 cell viability and expanded apoptosis and positive TUNEL cells. miR-506-3p upregulation augmented NSC-34 cell viability (Fig. 6f) and reduced positive TUNEL cells (Fig. 6g). The above findings demonstrated that miR-506-3p hampered the CCL2-CCR2 axis to attenuate LPS-induced microglial inflammation and IL-1β-elicited neuronal apoptosis.

### miR-506-3p Alleviated the Neuropathic Pain of BPA Rats

**Fig. 7:**
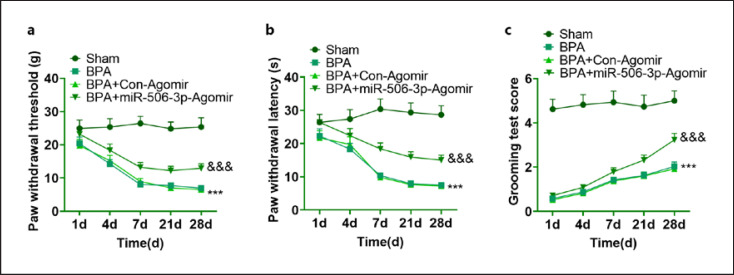
miR-506-3p relieved the neuropathic pain of BPA rats. The mechanical threshold (**a**) and the mechanical latency (**b**) of BPA rats quantified via von Frey (vF). **c** The motor function of the rats' upper limbs checked through TGT scoring. The statistics were represented as mean ± SD (*n* = 5). ***p < 0.001 (vs. Sham). ^&&&^*p <* 0.001 (vs. BPA + Con-Agomir).

To analyze the impact of miR-506-3p on BPA rats' pain, we conducted vF in all animal groups to quantify the mechanical hypersensitivity of the rats. From day 1 following surgery, in contrast with the Sham group, the mechanical threshold and the mechanical latency notably declined in BPA rats. miR-506-3p agomir was intraventricularly transfused into BPA rats. Then, we observed that the mechanical threshold and the pain threshold were dramatically heightened, suggesting that miR-506-3p mitigated mechanical pain threshold reduction induced by BPA (Fig. 7a, b). Moreover, we examined the motor function of the rats' upper limbs via TGT from day 1 to day 30 after surgery. By contrast to the Sham group, the rats in the BPA group developed lateral muscular atrophy and almost lost elbow flexion triggered by breathing movements. This phenomenon was especially conspicuous during the 2 weeks prior to surgery. However, it was slightly ameliorated during the 2–4 weeks subsequent to surgery with weak elbow flexion, and the TGT score was 0–2. Upon the impact of miR-506-3p on BPA rats, the degree of elbow flexion in BPA rats was greater. Such a phenomenon was more evident during the 2–3 weeks, and the TGT score was 2–3 (Fig. 7c). The above discoveries reflected that miR-506-3p relieved the neuropathic pain of BPA rats.

### miR-506-3p Lowered the Profiles of Inflammatory Factors and Suppressed Neuronal Apoptosis in the Spinal Cord Tissues of BPA Rats

**Fig. 8:**
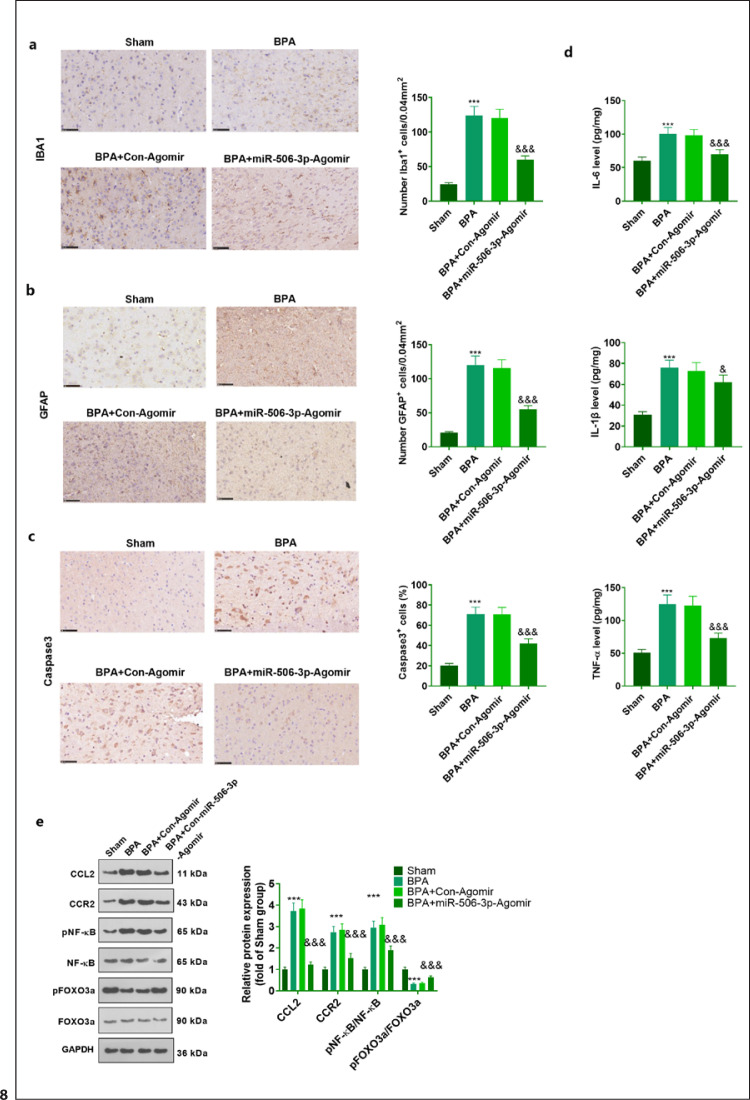
miR-506-3p attenuated the profiles of inflammatory factors and repressed neuronal apoptosis in BPA rats' spinal cord tissues. The positive cells of IBA1 (**a**), GFAP (**b**), and C-caspase-3 (**c**) in BPA rats' spinal cord tissues counted through IHC. **d** The levels of inflammatory cytokines in the spinal cord tissues verified by ELISA. **e** CCL2, CCR2, NF-κB, and FOXO3a profiles in the spinal cord tissues measured through Western blot. The statistics were presented as mean ± SD (*n* = 5). ***p < 0.001 (vs. Sham). ^&^*p <* 0.05, ^&&&^*p <* 0.001 (vs. BPA + Con-Agomir). IHC, immunohistochemistry.

Immunohistochemistry was carried out to check the number of positive GFAP, IBA1, and C-caspase-3 cells in the spinal cord tissues of BPA rats. It turned out that by contrast to the Sham group, the positive cells of GFAP, IBA1, and caspase-3 were increased in the rats' spinal cord tissues. miR-506-3p lessened the profiles of GFAP, IBA1, and caspase-3 in the spinal cord tissues of BPA rats (Fig. 8a-c). ELISA denoted that in contrast with the Sham group, the profiles of inflammatory factors (including TNF-α, IL-1β, and IL-6) were elevated in the spinal cord tissues, while miR-506-3p lowered their expressions (Fig. 8d). Western blot unraveled that in contrast with the Sham group, the protein profiles of CCL2 and CCR2 were upregulated, NF-κB activation was augmented, and FOXO3a activation was attenuated in the rats' spinal cord tissues. miR-506-3p reduced the protein profiles of CCL2 and CCR2, impeded NF-κB activation, and boosted FOXO3a activation (Fig. 8e). Overall, miR-506-3p exerts anti-inflammatory and antiapoptotic effects in BPA rat model.

## Discussion

BPA is known as a severe, disabling injury resulting from avulsion of BP. 30–80% of BPA patients may develop NP [32]. BP reimplantation can mitigate upper limb motor dysfunction in the wake of BPA. Nevertheless, it only has a limited function [2]. As reported, following BPA, Iba1-immunoreactive microglia and glia fibrillary acidic protein-immunoreactive astrocytes are remarkably initiated in the spinal cord tissues of BPA rats [5]. The activation of microglia and astrocytes following nerve damage is a pivotal participant in neuroinflammation, and the released inflammatory factors can elicit NP and chronic neuronal damage [33, 34]. Neurogliocyte activation inhibition can hinder NP elicited by BPA [35]. Here, we probed the influence of miR-506-3p on BPA-induced NP. We discovered that miR-506-3p expression was downregulated in the in vivo and in vitro BPA models, while miR-506-3p upregulation can impede the CCL2/CCR2/NF-κB axis to exert its neuroprotective and anti-inflammatory functions. Our research is the first to investigate the function of miR-506-3p in BPA, which may provide a novel target for BPA treatment.

Various miRNAs have been demonstrated to have dysregulated expressions in multiple nerve injuries. For instance, dysregulated miRNAs like miR-155 [36], miR-192-5p [37], miR-182-5p [38], and miR-30c [39] are closely associated with NP following nerve damage. More of note, in the BPA pain model, 10 miRNAs with different expressions have been uncovered in four different tissues [40], which has also been corroborated by Tang et al. [41]. miR-506-3p is a miRNA that is newly discovered. miR-506-3p overexpression dampens high glucose-elicited inflammation, oxidative stress, and apoptosis in HK-2 cells in high glucose-induced renal tubular epithelial cells [42]. This suggests that miR-506-3p boasts an anti-inflammatory function. Nevertheless, the function of miR-506-3p in BPA has not been reported so far. Thus, our research uncovered that miR-506-3p's expression was downregulated in the in vivo and in vitro BPA models. miR-506-3p upregulation impeded microglial inflammation and attenuated neuronal apoptosis while guarding against nerve damage in BPA rats, hence repressing NP elicited by BPA. Notwithstanding, the mechanism of miR-506-3p in BPA needs to be further explained in the following experiments.

Umpteen studies have denoted that miR-506-3p targets multiple mRNAs to exert their cancer-suppressing functions. For instance, miR-506-3p restrains GALNT4 expression to impede prostate cancer cell proliferation and metastasis [14]. miR-506-3p downregulates YAP1 expression at the mRNA and protein levels, suppressing papillary thyroid cancer cell proliferation [16]. Notwithstanding, the functions of miR-506-3p and CCL2 in BPA remain obscure. The ENCORI website demonstrated that there was a binding site between miR-506-3p and CCL2. Pearson analysis, dual luciferase assay, and RIP further substantiated the binding relationship between the two, which were negatively correlated. CCL2 upregulation augmented inflammatory factors in LPS-induced BV2 cells and IL-1β-elicited neuronal apoptosis, whereas miR-506-3p suppressed such an effect. Therefore, we conjectured that miR-506-3p impeded the CCL2/CCR2 signaling pathway to exert its anti-inflammatory and neuroprotective functions.

CCL2, a significant mediator of neuroinflammation, will be released and combine with CCR2 when the nervous system is damaged, hence recruiting inflammatory cells to damaged tissues [43]. Many studies have shown that following nerve injury, CCL2 expression is upregulated in injured dorsal root ganglion cells, and CCL2 upregulation can elicit microglial activation, hence incurring NP [44, 45]. Nuclear factor-κB (NF-κB), a prevalent transcription factor, can modulate inflammation in neurogliocytes [46]. In the spinal cord and dorsal root ganglion induced by BPA, the NF-κB signaling pathway is initiated, and NF-κB pathway activation inhibition can suppress BPA-elicited neuroinflammation and NP [35]. FOXO3a, a significant member of the forkhead transcription factor FOXO subfamily, partakes in important biological processes like cell apoptosis, proliferation, cell cycle progression, DNA damage, and tumorigenesis [47]. The KEGG database (https://www.kegg.jp/pathway/ko04062+K09408) indicates that CCL2 can boost NF-κB phosphorylation or dampen FOXO3 phosphorylation via CCR2 to mediate the survival and apoptosis of cytokines and cells. Prior studies have suggested that FOXO3a expression is downregulated in injured neurons [48]. The PI3K inhibitor LY294002 induced FOXO3a downregulation and bolstered the generation of TNF-α protein in LPS-elicited spinal cord cells [49]. Here, we discovered that in the in vivo and in vitro BPA models, the profiles of CCL2 and CCR2 were upregulated, and NF-κB and FOXO3a phosphorylation were augmented. Moreover, a targeted correlation existed between miR-506-3p and CCL2. miR-506-3p upregulation or downregulation stepped up or restrained CCL2 expression in the cells, while miR-506-3p impeded the boosting effect of CCL2 on microglial inflammation and neuronal apoptosis.

## Conclusion

We have demonstrated that miR-506-3p modulates the CCL2/CCR2/NF-κB axis to exert its neuroprotective and anti-inflammatory functions. Our findings have clarified that miR-506-3p is of potential value in BPA treatment, which may provide a new way of thinking for BPA.

## Statement of Ethics

This study protocol was reviewed and approved by Ethics Committee of the Affiliated Hospital of Beihua University (approval number [BU117027]).

## Conflict of Interest Statement

The authors declare that they have no competing interests.

## Funding Sources

This research did not receive any specific grant from funding agencies in the public, commercial, or not-for-profit sectors.

## Author Contributions

Conception and design of the experiments: Wei He; statistical analysis: Wei Zheng, Songyuan Chi, and Taihao Cui; writing the paper, methodology review, writing − review and editing, and performing the experiments: Xing Jin. All authors read and approved the final manuscript.

## Data Availability Statement

All data generated or analyzed during this study are included in this article. Further inquiries can be directed to the corresponding author.

## Figures and Tables

**Table 1:** Primers used for qRT-PCR analysis

Genes	Primer sequence(5′→3′)
miR-506-3p	
Forward primer	TAAGGCACCCTTCTGAGTAGA
Reverse primer	GCGAGCACAGAATTAATACGAC
IL-1β	
Forward primer	CGACAAAATACCTGTGGCCT
Reverse primer	TTCTTTGGGTATTGCTTGGG
IL-6	
Forward primer	GAAACCGCTATGAAGTTCCTCTCTG
Reverse primer	TGTTGGGAGTGGTATCCTCTGTGA
TNF-α	
Forward primer	CATCTTCTCAAAATTCGAGTGACAA
Reverse primer	TGGGAGTAGACAAGGTACAACCC
CCL2	
Forward primer	TCGGAGTTTGGGTTTGCTTG
Reverse primer	CAATCAATGCCCCAGTCACC
CCR2	
Forward primer	TCAGAGATGGCCAGGTTGAG
Reverse primer	ACGGTGCTCCCTGTCATAAA
GAPDH	
Forward primer	CTGGGGACGACATGGAGAAAA
Reverse primer	AAGGAAGGCTGGAAGAGTGC
U6	
Forward primer	CGCTTCGGCAGCACATATACTA
Reverse primer	CGCTTCACGAATTTGCGTGTCA
